# Evaluation of Artificial Intelligence as a Search Tool for Patients: Can ChatGPT-4 Provide Accurate Evidence-Based Orthodontic-Related Information?

**DOI:** 10.7759/cureus.65820

**Published:** 2024-07-31

**Authors:** Amani Alkhamees

**Affiliations:** 1 Department of Orthodontics and Pediatric Dentistry, College of Dentistry, Qassim University, Buraydah, SAU

**Keywords:** patient safety, reliability, orthodontics, chatgpt, artificial intelligence

## Abstract

Introduction: Artificial intelligence (AI) is already a part of our reality. Many people started using ChatGPT in their daily life, replacing existing web browsers. The confidence people put in the ability of ChatGPT to provide accurate medical information is increasing. With that, the need for proper assessment tools for the safety and reliability of ChatGPT is also crucial.

Objective: This study aimed to assess the accuracy, reliability, and quality of information provided by ChatGPT-4 on three specific orthodontic topics, namely, impacted canines, interceptive orthodontic treatment, and orthognathic surgery, as evaluated by five experienced orthodontists using a Likert scale ranking method.

Materials and methods: Using ChatGPT version 4, 20 most commonly asked questions were generated and answered on the following topics: impacted canines, interceptive treatment, and orthognathic surgery. The evaluation of the quality of the answers provided was done by five experienced orthodontists. Quality assessment was done using the Likert scale ranking method.

Results: The quality answers generated by a conversational AI system (ChatGPT4) were evaluated by five experienced orthodontists for three topics: impacted canines, interceptive orthodontics, and orthognathic surgery. The evaluators rated each question-answer pair on a five-point scale from "very poor" to "very good." The results showed that the AI system produced generally good quality information for all topics, with no significant difference between them. The inter-rater agreement among the experts was low, indicating some variability in their judgments.

Conclusion: This study demonstrates that ChatGPT4 can provide generally good information on impacted canines, interceptive treatment, and orthognathic surgery. However, answers provided should be handled with caution due to variability and lack of reliability and should not be considered a substitute for professional opinion.

## Introduction

The rapid changes in artificial intelligence (AI) technology are transforming many industries. AI refers to creating machines that mimic human intelligence, performing tasks such as visual perception, speech recognition, decision-making, and language translation [1]. Machine learning is a branch of AI that focuses on developing algorithms and statistical models that enable computer systems to learn from and improve their performance on specific tasks without being explicitly programmed [2]. On November 30, 2022, OpenAI (San Francisco, CA: OpenAI LP) launched its generative language model, ChatGPT (Chat Generative Pre-trained Transformer), allowing people to converse with a machine about various topics [3]. In January 2023, ChatGPT reached over 100 million users, making it the fastest-growing consumer application.

In the medical field, ChatGPT has proved its efficacy as a conversational agent or chatbot for patients. ChatGPT can generate natural language responses, making it ideal as a conversational agent interacting with patients in human language [4]. Another application is the ability to analyze large volumes of data, helping healthcare providers make more accurate diagnoses and improving patient safety [5]. ChatGPT may have vast applicability, and its use in medical specialties is of great importance.

ChatGPT has proven its ability to achieve a high accuracy rate passing the United States Medical Licensing Examination (USMLE) [6] and other medical examinations in specialty fields [7,8,9]. A need to emphasize that professionals with background knowledge have conducted all these studies needs to be brought to light. The genuine concern is with the increased use of patients of ChatGPT as a replacement for professional medical opinion. Previous use of traditional web browsers has always been taken with a grain of salt from patients and backed with additional medical visits to confirm searched information. The growing confidence in ChatGPT is concerning as it is based on the false belief of its genius ability to provide accurate information with no tangible proof yet.

Despite its potential, ChatGPT often produces seemingly credible but incorrect outputs, thus warranting caution when considering its applications in medical practice and research [10,11]. The reliability and accuracy of ChatGPT have yet to be evaluated enough, particularly in the context of subjective medical questions that patients are likely to ask. In the orthodontic field, it is known that treatment planning is a comprehensive, complex process that usually follows a subjective rather than objective pattern, being a case-sensitive and highly individualized protocol.

Information related to orthodontics topics provided by ChatGPT has yet to be evaluated in relation to its accuracy, reliability, and content validity. This is especially significant considering the increased use and trust of ChatGPT in patient's daily life. Subjective medical questions that patients are likely to ask regarding common orthodontic conditions and treatment options are now likely to be directed to ChatGPT.

This study aims to evaluate the safety and accuracy of ChatGPT-4 as a tool capable of providing evidence-based complete information for the patient regarding impacted canines, interceptive treatment, and orthognathic surgery.

## Materials and methods

Study design

Question Generation

Using ChatGPT-4, 20 frequently asked questions for each of the following topics were generated: impacted canines, interceptive orthodontic treatment, and orthognathic surgery. ChatGPT was prompted to generate the questions based on common patient inquiries in orthodontics .

Answer Generation

ChatGPT-4 provided answers to each of the 60 questions (20 per topic). All answers were saved and documented for further evaluation.

Evaluator Selection

Five experienced orthodontists with diverse clinical and educational backgrounds were selected as evaluators. Evaluators were provided with training to ensure they understood the scoring criteria.

Evaluation Criteria

Evaluators assessed the answers based on two main criteria: 1) accuracy: the extent to which the answer aligns with current scientific evidence, 2) comprehensiveness: whether the answer sufficiently provides necessary information for a layperson (parent, patient, etc.).

Scoring Method

Evaluators used a five-point Likert scale to rate each answer: 1 = very poor, 2 = poor, 3 = acceptable, 4 = good, and 5 = very good. Each evaluator independently rated the answers.

Data Collection

Ratings were recorded in a Microsoft Excel spreadsheet (Microsoft Corporation, USA) for analysis.

Statistical Analysis

Descriptive statistics (means, standard deviations, medians, and interquartile ranges) were calculated. Inter-rater reliability was assessed using Fleiss' Kappa statistics. Differences in topic ratings and evaluator ratings were analyzed using Kruskal-Wallis H tests. Confidence intervals and standard errors were computed to evaluate the precision of estimates.

## Results

A comprehensive expert assessment was conducted to evaluate the quality of orthodontic information covering three topics generated by a leading conversational AI system ChatGPT-4 (OpenAI, San Francisco, CA: OpenAI LP). Five orthodontists experts, each with profound experience in research, education, and clinical practice, evaluated a total of 60 AI-generated question-answer pairs, spanning three major orthodontics topics. The experts assigned a rating on a five-point Likert scale for each question-answer pair, with 1 being "very poor" and 5 being "very good" quality. Descriptive statistics for each question-answer pair rating are shown in Table 1.

**Table 1:** Descriptive statistics of the five-point Likert scale for the evaluators’ assessment of each question and answer in each topic

Topic	Question	Answer	Mean	SD	Median	IQR
1	Q1. What are impacted canines?	Impacted canines are teeth that have failed to emerge fully into their expected positions in the dental arch.	4.20	0.45	4.00	4.0-4.0
	Q2. How common are impacted canine teeth?	Impacted canines are relatively common, especially the upper canines.	3.20	1.10	3.00	3.0-3.0
	Q3. What causes canines to become impacted?	Causes include lack of space, early loss or retention of baby teeth, and abnormal growth.	3.60	1.14	4.00	3.0-4.0
	Q4. Are impacted canines painful?	They can be painful, especially if they cause crowding or cyst formation.	2.80	1.30	3.00	2.0-4.0
	Q5. How are impacted canines diagnosed?	Through dental examinations, X-rays, and sometimes CT scans.	3.80	1.30	4.00	3.0-5.0
	Q6. What are the treatment options for impacted canines?	Treatment options include orthodontic braces to open space, surgical exposure, and alignment of the impacted tooth.	3.40	0.89	4.00	3.0-4.0
	Q7. Is surgery always required for impacted canines?	Not always; it depends on the position and severity of the impaction.	4.40	0.55	4.00	4.0-5.0
	Q8. How long does treatment for an impacted canine take?	Treatment can take several months to a couple of years.	3.80	0.84	4.00	3.0-4.0
	Q9. Can impacted canines affect other teeth?	Yes, they can lead to crowding and displacement of adjacent teeth.	2.80	1.30	2.00	2.0-3.0
	Q10. What are the risks of not treating an impacted canine?	Risks include cyst formation, infection, and potential damage to adjacent teeth.	4.20	0.84	4.00	4.0-5.0
	Q11. At what age do impacted canines typically become noticeable?	They are often identified in early adolescence, around 12-13 years.	2.80	1.30	3.00	2.0-4.0
	Q12. Are there any signs or symptoms to watch for at home?	Look for delayed eruption of canines or misplaced teeth.	3.40	0.89	4.00	3.0-4.0
	Q13. How does impacted canine treatment differ in adults compared to children?	Treatment in adults may be more complex and take longer due to less bone flexibility.	3.40	1.34	4.00	2.0-4.0
	Q14. Can braces help in treating impacted canines?	Yes, braces are often used to create space for the impacted tooth.	4.20	0.84	4.00	4.0-5.0
	Q15. What is the success rate of impacted canine treatment?	Success rates are high, especially with early intervention.	4.60	0.55	5.00	4.0-5.0
	Q16. Are there any long-term effects of having an impacted canine?	Potential long-term effects include cyst formation and damage to adjacent teeth.	4.20	0.45	4.00	4.0-4.0
	Q17. How can I prevent my child from developing impacted canines?	Regular dental check-ups and early intervention can help.	4.80	0.45	5.00	5.0-5.0
	Q18. Does insurance typically cover treatment for impacted canines?	Coverage varies; many insurance plans cover part of the treatment.	3.20	1.64	4.00	2.0-4.0
	Q19. Can impacted canines reoccur after treatment?	Recurrence is rare if treated properly.	1.60	0.89	1.00	1.0-2.0
	Q20. Are there any alternative treatments to traditional methods for impacted canines?	Alternative treatments are limited; most cases require conventional orthodontic and surgical methods.	3.80	1.10	4.00	4.0-4.0
2	Q1. What is interceptive orthodontic treatment?	It's early intervention to address and prevent orthodontic issues in children.	4.60	0.55	5.00	4.0-5.0
	Q2. At what age should interceptive treatment begin?	Typically around age 7-9, based on individual needs.	4.00	1.00	4.00	3.0-5.0
	Q3. What dental issues can interceptive treatment address?	It addresses issues like crowding, jaw growth problems, and bite misalignments.	4.00	1.00	4.00	3.0-5.0
	Q4. How does interceptive treatment differ from traditional orthodontic treatment?	It starts earlier and focuses on guiding jaw growth and tooth position.	3.60	1.14	4.00	3.0-4.0
	Q5. What are the benefits of interceptive treatment?	Benefits include reduced need for future orthodontic treatment and better oral health outcomes.	4.40	0.89	5.00	4.0-5.0
	Q6. Are there any risks or downsides to interceptive treatment?	Risks are minimal but can include discomfort and the need for more treatment later.	3.60	0.89	3.00	3.0-4.0
	Q7. How long does interceptive treatment usually last?	Usually 1-3 years, depending on the case.	2.20	1.30	2.00	1.0-3.0
	Q8. Will my child need additional orthodontic treatment after interceptive treatment?	Some children might need additional treatment in their teens.	4.20	0.84	4.00	4.0-5.0
	Q9. What types of orthodontic appliances are used in interceptive treatment?	Appliances include expanders, space maintainers, and partial braces.	4.20	0.45	4.00	4.0-4.0
	Q10. How can I tell if my child might need interceptive treatment?	Look for signs like crowded or misplaced teeth, difficulty biting, or jaw misalignment.	3.80	0.84	4.00	3.0-4.0
	Q11. Is interceptive treatment painful?	There can be some discomfort, but it's usually minimal.	4.40	0.55	4.00	4.0-5.0
	Q12. How much does interceptive treatment cost, and is it covered by insurance?	Costs vary; many insurance plans offer partial coverage.	3.40	1.14	3.00	3.0-4.0
	Q13. Can interceptive treatment prevent the need for braces later on?	It can reduce but not always eliminate the need for future braces.	4.00	1.00	4.00	3.0-5.0
	Q14. What is the success rate of interceptive treatment?	Success rates are high, especially with early and appropriate intervention.	4.60	0.55	5.00	4.0-5.0
	Q15. How often will my child need to visit the orthodontist during interceptive treatment?	Regular visits every 6-8 weeks are typical for adjustments and monitoring.	3.00	1.22	3.00	2.00-3.0
	Q16. Are there any dietary restrictions during interceptive treatment?	Yes, avoiding hard, sticky, or chewy foods to protect the appliances.	4.20	0.84	4.00	4.0-5.0
	Q17. How do I care for my child’s orthodontic appliances?	Regular cleaning, avoiding certain foods, and following the orthodontist's instructions.	4.80	0.45	5.00	5.0-5.0
	Q18. Can interceptive treatment affect speech or eating?	Initially, there may be minor impacts on speech or eating, but these typically resolve quickly.	4.20	0.84	4.00	4.0-5.0
	Q19. What happens if an issue is identified but interceptive treatment is not pursued?	Delaying treatment can lead to more complex and lengthy treatments later.	4.60	0.55	5.00	4.0-5.0
	Q20. How does interceptive treatment impact overall dental health?	It can significantly improve long-term oral health by addressing problems early.	4.60	0.55	5.00	4.0-5.0
3	Q1. What is orthognathic surgery?	It's a surgical procedure to correct misalignments of the jaw and teeth.	4.00	0.71	4.00	4.0-4.0
	Q2. Who is a 4 candidate for orthognathic surgery?	Individuals with significant jaw misalignments that cannot be corrected with orthodontics alone.	4.00	1.22	4.00	4.0-5.0
	Q3. What are the benefits of undergoing orthognathic surgery?	Improved jaw function, facial appearance, and often speech and breathing.	4.80	0.45	5.00	5.0-5.0
	Q4. What are the risks associated with orthognathic surgery?	Risks include infection, bleeding, nerve damage, and need for further surgery.	4.60	0.55	5.00	4.0-5.0
	Q5. How is orthognathic surgery performed?	Through precise surgical cuts in the jawbones, realigning them into a more optimal position.	4.00	1.00	4.00	3.0-5.0
	Q6. How long does recovery from orthognathic surgery take?	Full recovery can take several weeks to a few months.	3.60	0.89	3.00	3.0-4.0
	Q7. Will orthognathic surgery change my appearance?	It can alter facial appearance, usually in a way that is more balanced and functional.	4.20	0.84	4.00	4.0-5.0
	Q8. How do I prepare for orthognathic surgery?	Preparation includes dental and medical evaluations, orthodontic treatment, and lifestyle adjustments for recovery.	4.40	0.55	4.00	4.0-5.0
	Q9. How long does orthognathic surgery typically take?	The surgery itself can take several hours, depending on complexity.	4.40	0.89	5.00	4.0-5.0
	Q10. What is the success rate of orthognathic surgery?	High success rates, especially when followed by appropriate post-operative care.	3.20	1.30	3.00	2.0-4.0
	Q11. Is orthognathic surgery painful?	Post-operative pain is manageable with medication and typically subsides within a few days.	4.20	0.84	4.00	4.0-5.0
	Q12. How much does orthognathic surgery cost, and is it covered by insurance?	Costs vary widely; many insurance plans provide coverage if deemed medically necessary.	3.60	1.34	3.00	3.0-5.0
	Q13. Will I need orthodontic treatment before or after surgery?	Often, pre-surgical orthodontic treatment is necessary, and sometimes post-surgical adjustments as well	3.40	1.14	3.00	3.0-4.0
	Q14. Can orthognathic surgery improve speech or chewing problems?	Yes, it often improves functional issues like speech and chewing.	4.20	0.84	4.00	4.0-5.0
	Q15. What kind of follow-up care is required after orthognathic surgery?	Regular follow-up visits, adherence to dietary restrictions, and careful oral hygiene.	3.60	0.89	3.00	3.0-4.0
	Q16. Are there any dietary restrictions after orthognathic surgery?	A soft or liquid diet is often recommended initially, gradually returning to normal eating.	4.20	0.84	4.00	4.0-5.0
	Q17. How long will I need to take off work or school after surgery?	Typically, 2-4 weeks off is recommended for recovery.	4.20	0.84	4.00	4.0-5.0
	Q18. What are the alternatives to orthognathic surgery?	Alternatives may include orthodontic treatment or dental appliances, depending on the severity of the issue.	3.60	0.89	3.00	3.0-4.0
	Q19. How can I choose the right surgeon for orthognathic surgery?	Look for a surgeon with experience in maxillofacial surgery and a 4 track record.	4.60	0.55	5.00	4.0-5.0
	Q20. Can orthognathic surgery correct TMJ disorders?	In some cases, it can alleviate TMJ symptoms, especially if they're related to jaw misalignment.	4.00	1.00	4.00	3.0-5.0

The overall mean rating given by the experts across all questions and topics was 3.89 (SD = 0.386, n = 300 ratings), indicating that the quality was generally rated as good (Table 2).

**Table 2:** Summary statistics for the overall rating of each evaluator and the difference between their ratings for all questions and answers

		ChatGPT evaluation											
		Very poor		Poor		Acceptable		Good		Very good		Mean (SD)	p-value
		N	N %	N	N %	N	N %	N	N %	N	N %		
Evaluator	1	3	5.0%	13	21.7%	8	13.3%	13	21.7%	23	38.3%	3.67 (0.321)	0.406
	2	0	0.0%	2	3.3%	19	31.7%	27	45.0%	12	20.0%	3.82 (0.257)	
	3	1	1.7%	4	6.7%	19	31.7%	34	56.7%	2	3.3%	3.53 (0.116)	
	4	3	5.0%	5	8.3%	10	16.7%	19	31.7%	23	38.3%	3.9 (0.36)	
	5	1	1.7%	1	1.7%	5	8.3%	11	18.3%	42	70.0%	4.53 (0.462)	
	Total	8	2.7%	25	8.3%	61	20.3%	104	34.7%	102	34.0%	3.89 (0.386)	

Kruskall-Wallis H test found no statistically significant difference between the mean ratings assigned by the five experts (p = 0.406). This may suggest that there were no detectable scoring biases among the experts and there was consensus in their evaluations.

Further analysis focused on assessing whether the AI system demonstrated consistent performance across the three assessed topics. The topics covered were impacted canines, interceptive orthodontics, and orthognathic surgery. No statistically significant difference was found between the mean expert ratings given to question-answer pairs belonging to each of the three topics (p = 0.368). The overall topic rating ranged from 3.61 to 4.04 on the five-point scale (Table 3), confirming that experts judged the quality of AI-generated information to be generally good, irrespective of the topic under assessment.

**Table 3:** Summary statistics for evaluator’s assessment and difference in the mean rating of the three topics

Topic	Mean	Standard deviation	Median	Cronbach’s alpha	p-value
1	3.61	0.279	4.0	0.354	0.368
2	4.02	0.471	4.0	0.874	
3	4.04	0.513	4.0	0.887	
Total	3.89	0.421	4.0	0.918	

The inter-rater agreement among the five experts was quantified using Cohen's kappa coefficient. The kappa coefficient gives a metric for how much homogeneity, or consensus, exists between two or more raters of categorical variables. The obtained kappa value was 0.104 (SEM = 0.006, 95% CI lower bound = 0.103, upper bound = 0.104) (Table 4), indicating "slight" or "poor" agreement according to the commonly cited benchmarks by Landis and Koch (1977).

**Table 4:** Agreement between different evaluators regarding their assessment using the five-point Likert scale

Rating category ^a^	Conditional probability	Kappa	Asymptotic			95% confidence interval	
			Standard error	z	Sig.	Lower bound	Upper bound
Very poor	0.027	-0.001	0.011	-0.120	0.904	-0.002	-0.001
Poor	0.083	0.050	0.011	4.667	0.000	0.049	0.050
Acceptable	0.203	0.041	0.011	3.878	0.000	0.041	0.042
Good	0.347	0.076	0.011	7.154	0.000	0.075	0.077
Very good	0.340	0.207	0.011	19.466	0.000	0.206	0.208
Overall agreement	-	0.104	0.006	16.165	0.000	0.103	0.104
a. Sample data contains five effective subjects and 60 ratings.							

This demonstrates an opportunity to calibrate rating standards through rater training and improved rubric development in order to obtain a strong consensus between experts for healthcare AI evaluation. Nonetheless, the aggregate data showed clear rating patterns, as only 2.7% of all question-answer pairs were rated as very poor while 68.7% were rated as good or very good (Table 2). This affirms the ability of the AI system to generate orthodontic information of generally good quality.

## Discussion

The primary purpose of the current study is to evaluate ChatGPT-4 as an advanced AI tool, particularly its safety and accuracy as a stand-alone tool capable of providing evidence-based correct and complete information for the patient regarding three major orthodontic topics: impacted canines, interceptive treatment, and orthognathic surgery. The most frequently asked questions regarding these topics to AI were generated and evaluated by expert professionals in the field. To date, there are few studies documenting the application and effectiveness of ChatGPT in orthodontics, making the findings of this study valuable and informative.

Since its impressive launch in November 2022, OpenAI’s ChatGPT has received massive responsiveness, owing to its human-like responses and vast knowledge in many fields. These features increased the use, trust, and dependability of ChatGPT despite the absence of concrete evidence of its validity [12].

In the last two years, various studies have been done on ChatGPT in an attempt to examine its performance across different health fields [5,13,14,15]. Two recent systematic reviews on ChatGPT were conducted. The first one evaluated the performance of ChatGPT in medical question-answering and concluded that ChatGPT exhibited an accuracy rate of 56% in addressing medical queries, with a higher percentage in specific fields (internal medicine 63%) [16]. The second systematic review assessed the potential of ChatGPT as a tool for medical and dental research and concluded that despite heterogeneity between studies included, ChatGPT shows promising potential in both fields of medicine and dentistry [17].

In orthodontics, AI performed well in certain areas, like cephalometric measurement. Companies like WebCeph, an FDA- and KFDA-approved AI-driven online orthodontic diagnostic software, are showing promising results [18]. A recent systematic review on AI-driven automated cephalometric landmark identification concluded that AI demonstrated positive results compared to manual tracing [19].

However, orthodontic treatment planning is not just based on cephalometric analysis. An orthodontic treatment plan aims to address the patient’s chief complaint, utilizing the patient’s history, clinical findings, and diagnostic measurement in customizing a tailored treatment plan. While that does not always align with the ideal option for the case, a lot of subjectivity is involved in treatment planning. Variability in decision-making between practitioners is also affected by many factors and is hugely influenced by personal clinical experience [20].

In attempting to make orthodontic treatment planning more of an objective process, many studies experimented with different algorithms, systems, and analyses [21,22,23]. A study by Peilin et al. proposed a multilayer perceptron artificial neural network to aid in predicting orthodontic treatment plans. The result of this study revealed that the neural network models could predict extraction and non-extraction cases with an accuracy rate of up to 94%. They also stated that the model can predict the anchorage plan and extraction pattern with 84.2% and 92.8% accuracy rates, respectively [24]. A 2021 systematic review on the performance of AI in orthodontics revealed that while these models performed remarkably well, helping to save time, simplifying procedures, and leading to a more efficient work process, they cannot be used as a substitute for an experienced orthodontist [25].

Most of these models are based on either artificial neural networks (ANNs) or convolutional neural networks (CNNs) [25]. Although they provide value to the orthodontist, they cannot be accessed or used by the common patient, unlike ChatGPT, which has almost replaced traditional search engines and gained global trust as a medical information provider. In this study, the results indicated that the quality of the answers provided by ChatGPT was generally rated as good or very good (68.7%). This result is also confirmed by Tanaka et al., who assessed ChatGPT answers on three topics, i.e., clear aligners, temporary anchorage devices, and digital imaging, and stated a majority of the content is considered very good (71%) [26].

Another observation is that AI demonstrated consistent performance irrespective of the topic under assessment, providing information on the studied three topics (impacted canines, interceptive orthodontics, and orthognathic surgery) of generally good quality. These results are similar to a study by Ebru et al., where they compared data quality, reliability, and readability of multiple AI-based chatbots in orthognathic surgery. Their results demonstrate that while ChatGPT displayed greater originality in providing answers, they had limited quality [27]. Another study evaluated the reliability and readability of ChatGPT-provided data on cleft lip and palate-related information. They concluded that although the information generated was of high quality, it was challenging to read, and they stressed the importance of professional assessment of this information [28].

It is important to note that even with the overall positive rating, the evaluators noted multiple observations about the nature of the answers provided by ChatGPT that warrant a special mention. These answers were unspecific, had limited precision, and tended to have a short general notion of discerption. While a professional can understand an incomplete answer on a deeper level thanks to his background, the patient will not be able to do so. ChatGPT also does not provide a reliable reference for the answer provided, lacks evidence-based information support, and increases the possibility of distribution of faulty information misconceptions and bias. It is important to remember that ChatGPT was trained on diverse datasets that included both scientific and false information found on the Internet [29]. Another point worth mentioning is that the current ChatGPT (version 4.0) model includes information until April 2023, so any breakthrough or new studies will not be noted in its answers. 

The inter-rater agreement among the five experts, quantified by Cohen's kappa coefficient, yielded a value of 0.104, indicating a "slight" or "poor" agreement according to Landis and Koch (1977). This low agreement highlights several critical issues: the inherent subjectivity of expert evaluations, the limited number of raters, and the complex nature of medical information. These factors contribute to the variability in assessments and suggest a need for standardized evaluation criteria to mitigate subjectivity. In an effort to enhance the accuracy of future studies, it is recommended to increase the number of experts involved and implement rater calibration sessions to align evaluation standards and reduce biases. In addition, exploring advanced statistical methods like Krippendorff's alpha could provide deeper insights into agreement levels. Understanding the training data and algorithms used by AI models like ChatGPT-4 may reveal sources of response variability and inform improvements in AI development. Incorporating these recommendations can lead to more reliable and consistent evaluations, ensuring that AI tools in healthcare function as valuable adjuncts to human expertise.

In this study, 11% of answers were rated poor or very poor; looking at the nature of these questions and answers, it was noticed that most of them concerned individual variability and needed a more precise answer. It was observed that ChatGPT does not mention if the topic in question is controversial or not, and particular emphasis is demanded on the incompleteness of the information provided. This was also observed in a study by Floyd et al., where ChatGPT failed to consistently generate accurate responses to the majority of radiation oncology patient-centered questions [14].

ChatGPT has only been available for a few years, but its impact and influence on people’s behavior can be sensed. While it is officially released as an AI language model, the common notion among the general population of users is that ChatGPT is a super-intelligent search engine that can provide you with valuable, trustworthy, and valid information in real time. That change in patient mindset could lead to the acquisition of false health information. Questions usually directed to the health practitioner like “What’s the treatment of my condition?” or “What’s the severity and consequences of this condition?” would be directed to ChatGPT instead. This shift could lead to the potential for misdiagnosis, lack of personalized care, and inadequate handling of complex medical conditions. Increased reliability on ChatGPT as a medical information provider could potentially lead to deleterious effects on patient safety. Spreading awareness among users regarding perceived information is essential.

While ChatGPT demonstrated an overall good knowledge of examined orthodontic topics, the variation among evaluated answers and evaluators’ notes provided prompts us to the sensitive nature and intricacy of orthodontic conditions. Whether it is an orthodontist or a patient-user, verification of information provided should be done using evidence-based, peer-reviewed studies. 

## Conclusions

This study was conducted to test the accuracy of ChatGPT-4-provided orthodontic information. The results demonstrated that ChatGPT-4 can provide generally good information on impacted canines, interceptive treatment, and orthognathic surgery. However, answers should be handled cautiously due to variability and lack of reliability. ChatGPT-4 still in its latest version is not capable of generating precise evidence-based complete information. ChatGPT-4 is not a substitute for professional opinion and should not be used to diagnose or treat orthodontic conditions. A need to raise awareness about the current limitations of ChatGPT-4 is warranted.
